# Modified-dropless protocol (nil intraocular) for micro-incision vitrectomy surgery (MIVS): a retrospective pilot study

**DOI:** 10.1186/s12886-023-02943-z

**Published:** 2023-05-04

**Authors:** Noraliz Garcia-O’Farrill, Gordon T. Brown, Allan A. Hunter

**Affiliations:** 1 Oregon Eye Consultants LLC, 3783 International Court, Suite 290, Springfield, OR 97477 USA; 2 Cascade Medical Research Institute LLC, 3783 International Court, Suite 290, Springfield, OR 97477 USA

**Keywords:** Dropless MIVS, Vitreoretinal surgery, Subconjunctival injections, Inflammation prophylaxis, Small gauge vitrectomy, Endophthalmitis

## Abstract

**Background:**

Perioperative infection and inflammation prophylaxis after ocular surgery has evolved over the years along with improvements in surgical equipment and a growing interest in alternatives to the standard topical eye drops. The purpose of this study is to evaluate the outcomes of a novel, modified-dropless protocol for 23-gauge (23-G), 25-gauge (25-G) and 27-gauge (27-G) micro-incision vitrectomy surgery (MIVS) that omits any intraocular injections of antibiotics or steroids.

**Methods:**

This Institutional Review Board-approved, single-surgeon retrospective study reviewed MIVS post-surgical outcomes in patients who received a modified-dropless protocol from February 2020 to March 2021. A total of 158 charts were reviewed, of which 150 eyes met the eligibility criteria. After each case, patients were administered a 0.5 cc subconjunctival injection of a 1:1 Cefazolin (50 mg/cc):Dexamethasone (10 mg/cc) in the inferior fornix and 0.5 cc of posterior Sub-Tenon’s Kenalog (STK). No intravitreal injections were administered, and no pre- or postoperative antibiotic or steroid eye drops were prescribed. For patients allergic to penicillin, separate subconjunctival injections of 0.25 cc each of Vancomycin (10 mg/cc) and Dexamethasone (10 mg/cc) were administered. The primary safety parameter was postoperative cases of endophthalmitis. Secondary endpoints consisted of Best-Corrected Distance Visual Acuity (BCVA), intraocular pressure (IOP), and postoperative complications (retinal detachments, inflammation, need for additional surgery) within three months of surgery. Statistical analysis was performed using chi-square (χ²) tests for categorical values, and a Student’s t-test to compare continuous outcomes.

**Results:**

The majority of surgeries (96%) were performed with the 27G MIVS platform. There were no cases of postoperative endophthalmitis. Mean logMAR BCVA improved from 0.71 (± 0.67) to 0.61 (± 0.60) post-operatively (p = 0.02). Excluding patients who had silicone oil tamponade, postoperative BCVA improved from 0.67 (± 0.66) to 0.54 (± 0.55) (p = 0.003). Mean IOP increased from 14.6 (± 3.8) to 15.3 (± 4.1) (p = 0.05). Ten patients required further medication therapy for an increase in IOP, one had inflammatory signs, and 14 required a second surgical intervention mostly due to recurrences of initial surgical indication.

**Conclusion:**

A modified-dropless postoperative protocol involving subconjunctival and posterior sub-Tenon’s injections only may be a safe and convenient alternative to topical eye drops for patients undergoing MIVS, but additional and larger studies are needed.

**Supplementary Information:**

The online version contains supplementary material available at 10.1186/s12886-023-02943-z.

## Background

Pars Plana Vitrectomy (PPV) has evolved since the initial development of a 17-gauge (17-G) (1.42 mm diameter) vitreous infusion suction cutter by Robert Machemer in 1970. Since then, and after the limitations involving 20-gauge (20-G) systems (i.e., wound leaks, gas leaks, hypotony, hemorrhagic choroidals), modern PPV has focused on smaller instrumentation. The current Microincision Vitrectomy Surgeries (MIVS) using 23-G, 25-G, and, since 2010, 27-G instruments have allowed sutureless self-sealing sclerotomies, improved maneuverability, reduced postoperative inflammation, fewer complications, and overall increased patient safety [1]. With the advent of similar smaller-incision techniques and subsequent improved outcomes and safety in cataract surgery, the standard postoperative regimen involving various topical eye drops also evolved to successful dropless protocols consisting of intracameral, intravitreal or subconjunctival antibiotics and corticosteroid injections at the end of surgery [2–4]. Standard eye drops regimens tend to be intricate leading to decreased patient compliance [5, 6].

The perioperative topical eye drops regimen following vitrectomy surgery has still to evolve to allow increased patient convenience and satisfaction without compromising safety. A dropless protocol after MIVS could allow the benefits seen after dropless cataract surgery with a focus on antimicrobial and anti-inflammatory efficacy postoperatively [2]. Although our group recently published a novel dropless protocol for MIVS, it included intravitreal medication injections. To our knowledge, this is the first study to evaluate the outcomes of a novel, modified protocol for MIVS that omits any intraocular injections or postoperative drops of antibiotics or steroids.

## Methods

This Institutional Review Board (IRB)-approved (PeaceHealth IRB #1,509,728) study used a retrospective approach to review MIVS post-surgical outcomes in patients who received a modified-dropless protocol from February 2020 to March 2021 at a single center (Oregon Eye Consultants, LLC). The research adhered to the tenets of the Declaration of Helsinki, and an informed consent waiver was granted by the IRB. Inclusion criteria consisted of any surgical indication for MIVS. Patients with a history of ocular trauma, complicated cataract surgery, younger than 18 years, lost to follow-up during the 3 month post-operative course, or those who had a peritomy at the moment of surgery were excluded from the study. A total of 158 charts were reviewed, of which 150 eyes from 143 subjects met the eligibility criteria.

All surgeries were performed by a single surgeon using standard surgical protocols for MIVS, including preoperative antisepsis with 5% povidone-iodine solution applied to eyelids and ocular surface. The modified-dropless protocol was performed at the conclusion of each case, as illustrated in Table 1. Patients were given a 0.5 cc subconjunctival injection of a combination (1:1) of Cefazolin (50 mg/cc):Dexamethasone (10 mg/cc) in the inferior fornix and a 0.5 cc of posterior sub-Tenon’s Kenalog (STK) injection. No intravitreal injections were administered, and no pre- or post-operative eye drops were prescribed. Patients who had a penicillin allergy (21 cases) followed an alternative protocol where they were instead given separate subconjunctival injections of 0.25 cc each of Vancomycin (10 mg/cc) and Dexamethasone, and the STK injection. Additionally, Maxitrol ointment (Neomycin, Polymyxin B and Dexamethasone) was applied over the ocular surface before covering the eye with a sterile pad and a shield on all patients at the end of surgery.

**Table 1:** Modified-dropless protocol

Subconjunctival injections	
Mixed 0.5 cc of 1:1	Cefazolin (50 mg/cc)
	Dexamethasone (10 mg/cc)
If Penicillin allergy: Separate 0.25 cc each of	Vancomycin (10 mg/cc)
	Dexamethasone (10 mg/cc)
**Posterior sub-Tenon’s injection**	
0.5 cc	Kenalog (Triamcinolone 40 mg/cc)
**Maxitrol ointment**	

Collected data from the electronic medical records included demography, past ocular history, and surgery, including gauge, tamponade, and surgical diagnosis. The primary safety parameter observed was postoperative cases of endophthalmitis within three months. Secondary efficacy outcomes consisted of pre- and postoperative comparisons of Best-Corrected Distance Visual Acuity (BCVA), intraocular pressure (IOP), and postoperative complications (retinal detachments, inflammation, need for additional surgery) within three months of surgery.

Statistical analysis was performed using Microsoft Excel. Results of continuous and categorical variables are presented as means (± standard deviation [SD]). A chi-square (χ²) test was used for categorical values, and a Student’s t-test was used to compare continuous outcomes, with a p-value of less than 0.05 representing statistical significance.

## Results

The study comprised 150 eyes from 75 females and 75 males (χ²=1) (mean age 70.3 ± 9.4; range 37–93) who underwent the modified-dropless protocol after MIVS, including 21 patients who underwent the alternative protocol due to penicillin allergies. Baseline characteristics are summarized in Table 2. Preoperatively, 64 eyes were phakic and 86 were pseudophakic (p = 0.07). Most of the surgeries were performed with the 27-G MIVS platform, the primary platform used by the surgeon: 144/150 cases with the 27-G, 2 cases with 25-G, and 4 cases using a combination of two 27-G and one 25-G port. The most common diagnoses included epiretinal membranes (ERM), vitreous floaters, and rhegmatogenous retinal detachments (RD).

**Table 2:** Baseline Characteristics

	Total (n = 150)
Age	70.3 ± 9.4
Gender	
Female Male	75 75
Lens status	
Phakic Pseudophakic	64 86
Gauge	
27G 25G 27/25G	144 2 4
Diagnoses	
Epiretinal membrane Floaters Rhegmatogenous RD Tractional RD Macular Hole Silicone Oil removal Vitreous Hemorrhage Vitreomacular Traction Retained Lens Fragment	48 31 28 12 10 8 6 5 1

RD = Retinal Detachment

Within the mean 70-days post-surgical period, there were no cases of postoperative endophthalmitis. Additionally, as shown in Table 3, mean logMAR BCVA from the sample had a statistically significant improvement from 0.71 (± 0.67) to 0.61 (± 0.60) postoperatively (p = 0.02) at the three-month timepoint evaluation (70.13 (± 36.99) days). Similarly, excluding patients who had silicone oil tamponade, postoperative BCVA improved from 0.67 (± 0.66) to 0.54 (± 0.55) (p = 0.003). Mean IOP showed a marginal but non-statistically significant increase from 14.6 (± 3.8) to 15.3 (± 4.1), at the three-month timepoint evaluation (70.13 (± 36.99) days) (p = 0.05). No intra-operative complications were reported. Regarding other postoperative complications, there were 10 cases (6.7%) with an increased IOP within the first two months after surgery. The elevated IOP ranged from 28 to 35 mmHg, with a maximum elevation of 58 mmHg in one subject. All cases were managed with pressure-lowering drops and three of them required anterior chamber paracentesis, resolving the increased IOP without further complications. One patient (0.7%) developed signs of inflammation (trace cells and posterior synechiae) in the operative eye that improved after topical steroid medication therapy. Fourteen subjects (9.3%) required a second surgical intervention within the 3-month window due to recurrent RDs (7/14), recurrent ERM (1/14), new or persistent macular holes (5/14), and vitreous hemorrhage (1/14).

**Table 3:** Comparison of baseline with post-operative outcomes

	Pre-Operative	Post-Operative (mean: 70 days)	p-value
Endophthalmitis	-	0	
Mean BCVA (logMAR)	0.71 ± 0.67	0.61 ± 0.60	**0.02**
Mean BCVA excluding silicone oil	0.67 ± 0.66	0.54 ± 0.55	**0.003**
Mean IOP (mmHg)	14.6 ± 3.8	15.3 ± 4.1	0.05

Bold values statistically significant

## Discussion and conclusion

The modified-dropless protocol described in this study omitting any intraocular drug injections may be a safe alternative to the current standard post-surgical topical eye drops for patients undergoing MIVS. There were no cases of postoperative endophthalmitis or signs of infection within the postoperative period. Endophthalmitis is a concerning postoperative outcome, but studies have shown the incidence after MIVS is very low, ranging from 0.012–0.11% [7–9]. Zafar et al. observed a higher risk of endophthalmitis in patients aged more than 75 years, undergoing combined cataract and retina or glaucoma surgery, and those with a Charlson Comorbidity Index greater than zero [10]. The average age of our patient population (70.3 ± 9.4) is within the increased risk of post-surgical endophthalmitis but given the small sample size any definitive conclusion would be dependent on larger studies with sufficient power than this proof-of-concept study.

There was a statistically significant improvement in mean postoperative BCVA in our study, which was more significant excluding subjects with silicone oil tamponade. A non-statistically significant increase in IOP was maintained within the normal range, except in 6.7% of cases where additional medication treatment was required to lower pressures without further complications. Most of these patients requiring further management to lower IOP had silicone oil or gas tamponade with either sulfurhexafluoride (SF6) or perfluoropropane (C3F8). This is similar to the reported range of 0.7 to 10.2% of raised IOP after vitrectomy surgery, with a higher incidence in patients with gas or silicone oil tamponade [11, 12]. However, an increase in IOP secondary to the triamcinolone injection cannot be excluded. A second surgical intervention within the 3-month follow-up was only required in 9.3% of subjects due to recurrences of the initial surgical indication, new-onset macular holes, or vitreous hemorrhages.

Prophylactic regimens to reduce perioperative infection and inflammation should aim to initially limit organism entry into the eye and subsequently eliminate those that gain access. As widely shown in the literature, the only definitively proven agent to decrease the risk of endophthalmitis and initially achieve the first goal of limiting organism entry is the use of pre-operative povidone iodine by reducing bacterial growth [13]. For the latter goal, an array of antibiotics and delivery methods have been published, without a consensus on the best method to be used prophylactically. The dropless protocol aims to provide a more intrinsic anti-inflammatory effect and infection prophylaxis and eliminate patient compliance concerns. More so, a dropless alternative would diminish current challenges with eye drop efficacy, including uncertain ocular penetration and decreased residual volume after instillation in a conjunctival sac with common postoperative ocular changes after vitrectomy (e.g., conjunctival chemosis and eyelid swelling). Kang et al. demonstrated a significant decrease in post-MIVS eye drop instillation residual volume that continues until postoperative day four, especially with postoperative chemosis [14]. Subconjunctival injections provide a higher permeability through the retina and choroidal area than the topical route and are less invasive than intravitreal ones [15, 16]. Repeated short courses of prophylactic topical antibiotics may also add to the emergence of resistant bacterial strains and limit future management of ocular infections, with a single short course being enough to create a selection bias for resistance [17–19]. Kaldirim et al. reported a development of ocular surface organisms with resistance to fluoroquinolones in patients with diabetic macular edema that received frequent short courses of this topical antibiotic after intravitreal injections [17]. Therefore, the described modified-dropless protocol with subconjunctival antibiotics injection could be a promising alternative to the topical route in reducing postoperative endophthalmitis.

As modern ophthalmic surgery evolves towards smaller instrumentation and fewer complications, the authors believe peri-surgical management of infection and inflammation could also improve to maximize surgical outcomes, clinical practice efficacy, and patient satisfaction. Topical regimens have been associated with decreased patient compliance as they administer drops suboptimally which may compromise safety, prolong inflammation, and delay healing [6]. Eye drop self-administration obstacles have also been reported due to physical limitations and preservatives discomfort. Therefore, administration of topical prophylaxis may be handed to caregivers which also translates to a burden to them as they adjust their personal schedule to accommodate the post-operative needs of their relatives, including post-surgical transportation to the pharmacy to acquire the medications promptly [6, 20, 21]. During this study, physician-administered injections at the end of surgeries has decreased the burden to patients and caregivers, particularly in the elderly population, by removing the need for drop administration, eliminating drop-related discomfort, and reducing costs with only a $5 cost per patient that the surgical center covers as part of the total standardized current procedural terminology (CPT) surgery center fee.

The author group recently published a successful dropless protocol with additional 0.1 cc intravitreal injections of the combined 1:1 Cefazolin:Dexamethasone [22]. The modified protocol in the current study was designed to omit intravitreal injections, with similar efficacy as anti-inflammatory and infection prophylaxis, while aiming to improve patient safety. Injections have inherent risks and many intravitreal antibiotics have been associated with adverse effects. Intravitreal vancomycin has a well-established association with hemorrhagic occlusive retinal vasculitis (HORV) and non-hemorrhagic vasculitis resulting in severe visual loss after its use as treatment or prophylaxis for suspected endophthalmitis after intraocular surgery [23–25]. Although the pathogenicity of HORV is not fully understood, it is believed to be attributed to a delayed type-III hypersensitivity reaction rather than a toxic reaction to vancomycin [23]. Therefore, with avoidance of intravitreal injections, omitting topical eye drop logistics, and potentially selecting cases with low-risk characteristics (less than 65 years old, uncomplicated surgeries, low Charlson Comorbidity Index), patients may benefit from the modified-dropless protocol.

Although this peri-operative prophylaxis option seems to have good potential, our study presents some limitations. First, it was a single-center, single-study, retrospective study using mostly 27-G with neither controls nor a comparative group, which may have introduced confounding variables, and restricts the generalizability of results in comparison to the standard topical drops regimen, to other vitrectomy systems and to other surgical centers. Also, a larger cohort may be needed to estimate the risk of endophthalmitis or other complications.

In conclusion, we have now published the results of over 600 patients with both studies combined. This study further suggests that a modified-dropless postoperative protocol involving subconjunctival and posterior sub-Tenon’s injections only, may be a safe and convenient alternative to topical eye drops for patients undergoing MIVS, but additional and larger studies are needed.

## Electronic supplementary material

Below is the link to the electronic supplementary material.

Supplementary Material 1

## Data Availability

The datasets used and/or analyzed during the current study are available from the corresponding author on reasonable request.

## List of abbreviations

BCVA: Best-corrected visual acuity
CPT: Current procedural terminology
C3F8: Perfluoropropane
ERM: Epiretinal membrane
-G: Gauge
HORV: Hemorrhagic occlusive retinal vasculitis
IOP: Intraocular pressure
MIVS: Micro-incision vitrectomy surgery
PPV: Pars plana vitrectomy
RD: Retinal detachment
STK: Sub-Tenon’s Kenalog
