# Supplementary material for: Modified-dropless protocol (nil intraocular) for micro-incision vitrectomy surgery (MIVS): a retrospective pilot study
Source: BMC Ophthalmol. 2023 May 4;23:195. doi: 10.1186/s12886-023-02943-z (PMC10157951; doi:10.1186/s12886-023-02943-z)
Supplement: Supplementary file 1 — Supplementary Material 1 [file 12886_2023_2943_MOESM1_ESM.docx]

**Supplemental Table 1.** Intraoperative Characteristics

|  | Total  (n=150) |
| --- | --- |
| Tamponade  None  AFx  SF6  C3F8  SO | 22  90  13  13  12 |
|  |  |
|  |  |
| Additional Treatments  None  Endolaser  Cryotherapy  Combined | 96  27  17  10 |
|  |  |
|  |  |

AFx = Air-Fluid exchange; SF6 = Sulfurhexfluoride; C3F8 = Perfluoropropane; SO = Silicone oil

**Supplemental Table 2.** Patients who underwent repeat surgery

| Subjects | Initial surgery | Tamponade | Reason for repeat surgery |
| --- | --- | --- | --- |
| 1 | RD | C3F8 | Recurrent RD |
| 2 | SO removal | C3F8 | ERM |
| 3 | Tractional RD, VMT | AFx | Macular hole |
| 4 | RD | C3F8 | Recurrent RD |
| 5 | ERM | AFx | Recurrence of previous RD |
| 6 | Tractional RD, PDR with edema | AFx | Recurrent RD |
| 7 | MH | SF6 | Recurrent MH |
| 8 | Complex RD, PVR | SO | Recurrent RD |
| 9 | MH | SF6 | Recurrent MH |
| 10 | RD with multiple defects | C3F8 | Recurrent RD |
| 11 | SO removal | AFx | Recurrence of previous RD |
| 12 | Vitreous floaters | None | VH |
| 13 | ERM | AFx | Macular hole |
| 14 | RD with multiple defects | SF6 | Macular hole |

RD = Retinal detachment; AFx = Air-Fluid exchange; SF6 = Sulfurhexfluoride; C3F8 = Perfluoropropane; SO = Silicone oil
